# Impact of Impaired Fasting Glucose on Musculoskeletal Pain Among Female-Dominated Healthcare Workers

**DOI:** 10.3390/jpm15040122

**Published:** 2025-03-23

**Authors:** Yong-Hsin Chen, Jia-June Lin, Hsiu-Mei Tang, Ching-Wen Yang, Gwo-Ping Jong, Yi-Sun Yang

**Affiliations:** 1 Department of Health Policy and Management, Chung Shan Medical University, Taichung 402, Taiwan; a6328539@gmail.com; 2 Department of Public Health, Chung Shan Medical University, Taichung 402, Taiwan; 3 Department of Occupational Safety and Health, Chung Shan Medical University Hospital, Taichung 402, Taiwan; 4 Nursing Department, Chung Shan Medical University Hospital, Taichung 402, Taiwan; 5 Department of Internal Medicine, Chung Shan Medical University Hospital, Taichung 402, Taiwan; 6 Institute of Medicine, Chung Shan Medical University, Taichung 402, Taiwan; 7 Department of Endocrinology and Metabolism, Chung Shan Medical University Hospital, Taichung 402, Taiwan; 8 School of Medicine, Chung Shan Medical University, Taichung 402, Taiwan

**Keywords:** diabetes, prediabetes, impaired fasting glucose, musculoskeletal pain, overweight, obese

## Abstract

**Introduction**: In 2021, 10.5% of people aged 20–79 had diabetes, projected to rise to 12.2% by 2045, causing early deaths and straining healthcare systems. Musculoskeletal (MS) pain is common, affecting many workers and the general population. Prediabetes, notably impaired fasting glucose (IFG), is linked to increased MS pain risk. Objective: This study aims to assess IFG’s impact on MS pain and specific pain sites to aid prevention strategies. **Methods**: This cross-sectional study used the ‘2023 Employee Occupational Safety and Health Management Database’ from a Taichung hospital. It included health checks, demographics, living and work data, and MS pain surveys. Out of 2369 staff members contacted, 1039 valid responses were analyzed, excluding incomplete data, diabetes history, or fasting blood glucose levels above 125 mg/dL. Data on sex, age, marital status, coffee and alcohol consumption, sleep duration, exercise habits, height, weight, chronic diseases, profession, work hours, shift work, and education level were collected. Fasting blood glucose was verified using American Diabetes Association criteria (100–125 mg/dL). The Nordic Musculoskeletal Questionnaire (NMQ) measured MS pain frequency and severity, creating a pain degree index. **Results**: Overall, 21.17% had IFG. Participants were mostly female (85.18%), averaging 37.50 years. Neck and shoulder pain risk was linked to sex, coffee and alcohol consumption, sleep, exercise, chronic diseases, work hours, and IFG. Ankle pain risk was linked to coffee and alcohol consumption. IFG, coffee, alcohol, sleep under 6 h, chronic diseases, and work hours were independent risk factors for neck and shoulder pain. IFG was a risk factor for those without overweight or obesity. A mediation model tested IFG’s indirect effect on neck and shoulder pain among overweight or obese individuals, showing that IFG mediates the relationship between being overweight or obese and increased neck and shoulder pain risk. **Conclusions**: Among female-dominated healthcare workers, IFG, daily coffee, recent alcohol consumption, less than 6 h of sleep, chronic diseases (excluding diabetes), and longer work hours are independent risk factors for neck and shoulder pain. IFG mainly affects these areas, increasing pain risk regardless of body weight. Healthy blood glucose levels are associated with a lack of musculoskeletal pain, suggesting a novel prevention approach needing further study.

## 1. Introduction

In 2021, 10.5% (536.6 million) of people aged 20–79 had diabetes, a figure that is projected to increase to 12.2% (783.2 million) by 2045 [1]. Diabetes causes early death (46.6% of deaths in adults under 60 [2]) and strains healthcare systems. Taiwan has over 2 million diabetics, which is increasing by 25,000 annually (Health Promotion Administration). Unfortunately, globally, 44.7% of adult diabetics are unaware of their diabetic states [3]. Only around 5–10% of prediabetics develop diabetes annually [4] as restoring normal blood sugar levels during the prediabetic or early type 2 diabetes stages can prevent progression [5]; that is, the transition from being prediabetic to diabetic can be reversed through intervention. For instance, some studies have also shown that diet and/or exercise can effectively reduce the progression from prediabetes to diabetes [6,7].

Musculoskeletal (MS) pain is a common condition. For instance, MS pain prevalence is over 40% for professional drivers [8] and over 60% for academic staff [9], dentists [10], and healthcare providers working in the operating room [11]. Continued MS pain can lead to disability, reduced work capability, and lost wages [12]. MS pain is also common in the general population. In the United States, more than one-third of the population experiences MS pain, contributing to an economic burden of approximately USD 380.9 billion per year [13]. Common MS pain sites are the lower back, shoulder, and neck [14], studies of which have identified several possible risk factors: sex [15], long work hours [16], occupational stress [17], alcohol consumption [18], and sleep duration [19]. Notably, a close relationship between MS pain and chronic disease had been found. A meta-analysis study revealed that the prevalence of cardiovascular disease in people with MS pain was nearly twice as high as in those without MS pain [20], and a cross-sectional study indicated that type 2 diabetes was associated with chronic neck/shoulder pain [21]. An animal study demonstrated that hyperglycemic conditions induce painful symptoms; however, these can be reversed if the blood glucose is normalized [22,23]. Another study concluded that the MS pain resulting from type 2 diabetes could be due to the buildup of glycation end products in connective tissues and joints, particularly in the neck, knees, hips, shoulders, back, and arms [24].

MS pain could be linked to obesity. For instance, shoulder pain may be associated with obesity [25]. Veterans with MS pain frequently have higher obesity rates [26]. Obesity is characterized by low-grade inflammation [27], which is also linked to type 2 diabetes [28]. Given that obesity has many causes [29], whether individuals who are overweight or obese and have prediabetes are at a higher risk for MS pain must be confirmed through further assessment.

According to the above research, prediabetes could be associated with an increased risk of MS pain. Prediabetes is a stage between normal glucose tolerance and type 2 diabetes, including impaired glucose tolerance and impaired fasting glucose (IFG) [30]. A significant number of patients newly diagnosed with IFG develop type 2 diabetes within three years [31]. Additionally, IFG screening is generally simpler and less costly because it involves a single fasting blood glucose test. In contrast, impaired glucose tolerance screening requires an oral glucose tolerance test, which is more time-consuming and expensive [32]. Based on the above reasons, this study adopted IFG to assess whether the condition of IFG worsens MS pain and to identify specific pain sites associated with IFG for healthcare employees. It also seeks to confirm whether overweight or obese healthcare employees have a higher risk of MS pain due to prediabetes. The findings will aid in developing effective prevention strategies for individuals.

## 2. Materials and Methods

### 2.1. Study Design

This cross-sectional study utilized the ‘2023 Employee Occupational Safety and Health Management Database’ from a hospital affiliated with a medical university in Taichung, Taiwan, which was approved for use by the institutional review board (IRB) through an expedited review process (No: CS2-24164). It included employee health checks, demographic variables, living and work data, and MS pain surveys. In 2023, there were a total of 2369 employees with at least one year of service. After excluding samples with missing data, no health check reports, a history of diabetes, or fasting blood glucose levels exceeding 125 mg/dL, of the 1630 responses received, 1039 were deemed valid.

### 2.2. Participant Information: Demographics, Lifestyle, and Occupational Data

The questionnaire offered two sex options: female and male. Participants also provided their age. For marital status, the available choices were married and other. The response options for coffee consumption were never, occasionally, one cup per day, two cups per day, and at least two cups per day. For alcohol use in the past month, the options were as follows: never, occasionally, and daily. Additionally, daily sleep duration was surveyed with the following response options: less than 5 h, 5–6 h, 6–7 h, 7–8 h, and more than 8 h. The participants’ exercise habits were assessed using the following options: never, less than once a month, at least once a month, at least once a week, and at least once a day, which were, respectively, scored as 0, 25, 50, 75, and 100 points.

Height and weight were classified according to the definitions provided by the Health Promotion Administration, Ministry of Health and Welfare, Taiwan (https://www.hpa.gov.tw/Pages/List.aspx?nodeid=1757, accessed on 12 December 2024): underweight (Body Mass Index (BMI) < 18.5), healthy weight (18.5 ≤ BMI < 24.0), overweight (24.0 ≤ BMI < 27.0), and obese (BMI ≥ 27.0). Participants ticked relisted chronic diseases, with one or more diseases indicating that they were “suffering from chronic disease”. The professional field response options included nurses, administrative staff, physicians (attending physicians, residents, and nurse practitioners), and technical staff. Participants self-reported their daily work hours. For shift work, the response options were irregular, regular, night, and day shifts. The response options for education level were PhD, master’s, bachelor’s, and other.

### 2.3. Fasting Blood Glucose Data

Fasting blood glucose levels were verified by the laboratory of a hospital affiliated with a medical university. In 2003, the American Diabetes Association updated the diagnostic criteria for impaired fasting glycemia, expanding the fasting blood glucose range from 110–125 to 100–125 mg/dL [33]. We used the new criteria (100–125 mg/dL) to define prediabetes, which is now referred to as impaired fasting glucose (IFG).

### 2.4. Musculoskeletal Pain Measurement

The Nordic Musculoskeletal Questionnaire (NMQ) was used to measure the presence of MS pain in the preceding year. NMQ has been widely used worldwide as a repeatable, sensitive, and reliable pain measure [34,35,36]; for example, when tested against clinical histories, the effectiveness results differed by less than 20% [37]. The possible NMQ pain sites included the neck, shoulders, upper back, waist/lower back, elbows, wrists, hips/thighs/buttocks, knees, and ankles, with the pain frequency options being every day, once a week, once a month, once every six months, and at least once every six months (respectively, scored as 100, 80, 60, 40, and 20 points). Serious MS pain options were “life affected”, “need leave to recuperate”, “work ability significantly reduced”, “slightly reduced work capacity”, and “no effect on life and work” (respectively, scored as 100, 75, 50, 25, and 5 points). We used the product of the frequency and seriousness scores as the pain degree index. Risk is generally defined as a combination of the probability and severity of adverse effects [38] or consequences [39]; therefore, in this study, we call the pain degree index MS pain risk.

### 2.5. Research Procedures and Statistical Methods

To achieve our research objectives, we will employ a series of statistical methods. The steps undertaken are outlined below.

Step 1: The NMQ included detailed information on pain sites and frequency, which complicated the statistical analyses. Therefore, we used factor analysis to identify new variables to improve the interpretation. Varimax rotation provided standardized scoring coefficients and new factor loadings. As outlined in Hair et al. [40], factors with eigenvalues greater than 1 were retained.

Step 2: We described the demographic and survey variables for the 1039 participants. Additionally, we used *t*-tests or one-way ANOVA to identify the confounders of MS pain.

Step 3: A multiple linear regression model would be established to confirm if IFG is a independent risk factor for MS pain. Additionally, we would conduct stratified analyses to determine if overweight or obese individuals with IFG face the same increased risk of musculoskeletal pain as those with IFG who are of healthy weight.

Step 4: If the effect of IFG on MS pain was not significant among individuals who are overweight/obese or those with a healthy weight/underweight, mediation analysis would be used to further assess the relationship between IFG and MS pain. For this analysis, we followed the four strategies proposed by Baron and Kenny [40] and the revised suggestions by Shrout and Bolger [41]: (1) the first-stage effect: the independent variable significantly affects the mediating factor; (2) the independent variable significantly affects the dependent variable when the mediating factor is absent; (3) the second-stage effect: the mediating factor has a significant unique effect on the dependent variable; and, (4) the effect of the independent variable on the dependent variable weakens when the mediating factor is added to the model. Note that item (2) is recommended but not required. According to Iacobucci (2012) [42], if the mediating factor or dependent variable is a categorical variable, the Sobel test formula is rederived.

$$Z_{mediation\;}\left(Z_{m}\right)=\frac{\frac{a}{s_{a}}\times \frac{b}{s_{b}}}{\sqrt{{\;(\frac{a}{s_{a}})}^{2}+({\frac{b}{s_{b}})}^{2}+1}}$$

where a represents the linear regression coefficient of the independent variable against the mediating factor, and b represents the linear regression coefficient of the mediating factor against the dependent variable. The standard errors for a and b are, respectively, denoted $s_{a}$ and $s_{b}$, and $Z_{m}$ exceeding |1.96|, |2.57|, and |3.90| (for a two-tailed test) was, respectively, deemed significant at α = 0.05, 0.01, and 0.0001.

The analysis was conducted using SAS Enterprise Guide 7.1 software (SAS Institute Inc., Cary, NC, USA), with the significance defined as *p* < 0.05.

## 3. Results

Table 1 shows MS pain site and factor analysis. Following the principle proposed by Hair and Anderson (1995) [40], the eigenvalues for Factors 1 and 2, which were 6.06 and 1.83, respectively, were retained as they both exceeded 1. The factor loadings were then converted into standardized scoring coefficients using the varimax rotation method. The relatively high factor loading values for Factors 1 and 2, respectively, corresponded to the MS pain sites of the neck, shoulders, and ankles. Based on this, we defined Factor 1 as pain in the neck and both shoulders and Factor 2 as pain in both ankles. The cumulative explained variance for these two factors reached 83.16% in this NMQ. These redefined factors would substantially support our subsequent analysis.

Table 2 indicates that 21.17% of the participants had IFG. The majority of the participants were female (85.18%), with an average age of 37.50 ± 9.95 years. The participants were composed of physicians (11.65%), nurses (45.72%), technical staff (9.82%), and administrative staff or others (32.82%). Regarding MS pain risk, sex (*p* = 0.018), coffee drinking habits (*p* = 0.014), alcohol use in the past month (*p* < 0.001), daily sleeping time (*p* = 0.008), exercise habits (*p* = 0.024), chronic diseases (excluding diabetes) (*p* < 0.001), and work hours (*p* = 0.005) were associated with the risk of neck and shoulder pain. Additionally, coffee drinking habits (*p* = 0.011) and alcohol use in the past month (*p* = 0.002) were also associated with the risk of ankle pain. Remarkably, only the risk of neck and shoulder pain was associated with IFG (*p* = 0.030). Next, we will focus on the relationship between the risk of neck and shoulder pain and IFG.

Based on statistical validity, we integrated the response options for ‘Drinking coffee habits’—‘one cup per day’, ‘two cups per day’, and ‘at least two cups per day’—into ‘at least one cup per day’. The response options for ‘Alcohol use in the past month’—‘occasionally’ and ‘drinking every day’—were integrated into ‘ever’. The response options for ‘Daily sleeping time’—‘<5 h’ and ‘5–6 h’—were combined into ‘≤6 h’, while ‘6–7 h’, ‘7–8 h’, and ‘>8 h’ were combined into ‘>6 h’. Moreover, the response options for ‘Exercise habits’—‘at least once a day’ and ‘at least once a week’—were integrated into ‘at least once a week’; ‘at least once a month’, ‘less than once a month’, and ‘never’ were integrated into ‘less than once a week’. Table 3 indicated that among all participants, IFG (B = 0.19, *p* = 0.005), consuming at least one cup of coffee per day (B = 0.13, *p* = 0.03), ever drinking in the past month (B = 0.23, *p* < 0.001), daily sleep time of ≤6 h (B = 0.14, *p* = 0.020), suffering from chronic diseases (excluding diabetes) (B = 0.20, *p* < 0.001), and daily work hours (B= 0.08, *p* = 0.017) were independent risk factors for risk of neck and shoulder pain. Remarkably, among individuals without overweight or obesity (OW/OB), IFG was an independent risk factor for neck and shoulder pain (B = 0.29, *p* = 0.003), but it was not among individuals with OW/OB (B = 0.08, *p* = 0.444).

**Figure 1:**
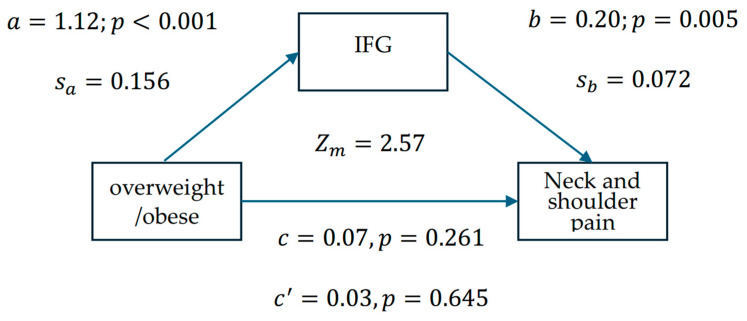
Mediation model examining the statistical associations between overweight/obesity, IFG, and neck and shoulder pain risk among 1039 healthcare workers, in which a represents the logistic regression coefficient for overweight/obese against IFG, and b represents the linear regression coefficient for IFG against the risk of neck and shoulder pain. The standard errors for a and b are denoted $s_{a}$ and $s_{b}$. The Sobel test (Z_m_ = 2.57, *p* = 0.01) indicates a significant indirect association via IFG, though causality cannot be inferred from these cross-sectional data.

Since we did not find that IFG impacts the increased risk of neck and shoulder pain among individuals with OW/OB, we utilized a mediation model (Figure 1) to test the indirect effect of IFG on neck and shoulder pain. Figure 1 shows that the first- and second-stage effects were statistically significant (a = 1.12, *p* < 0.001; b = 0.20, *p* = 0.005). The overweight or obese effect on the risk of neck and shoulder pain weakened when the mediating factor, IFG, was added to the model (from c = 0.07 to c’ = 0.03). The Sobel test (rederived according to Iacobucci) was used to determine if IFG mediated the relationship between being overweight or obese and the risk of neck and shoulder pain. The results suggested that IFG is a mediating factor between overweight or obese and the risk of neck and shoulder pain (Z_m_ = 2.57, *p* = 0.01). Notably, the direct effect was not significant (c’ = 0.03, *p* = 0.645), indicating a fully mediating effect.

## 4. Discussion

We found in multiple linear regression that IFG, consuming at least one cup of coffee per day, drinking alcohol in the past month, having a daily sleep time of less than 6 h, suffering from chronic diseases (excluding diabetes), and increased daily work hours were associated with neck and shoulder pain. The results support the study’s aim by showing that IFG is associated with the presence of musculoskeletal pain, specifically in the neck and shoulder.

In addition to the lower and upper back, the neck and shoulders were predominant musculoskeletal pain sites in the healthcare worker sample [43]. In comparison with the research presented in Table 1, the neck and shoulders were also the primary pain sites among participants. The IFG prevalence in the participant sample was 21.17%. In comparison, the prediabetes prevalence in Taiwanese adults aged 18 and older from 2017 to 2020 was 25.50% [44]. A study indicated that relying solely on fasting glucose levels may lead to an underestimation of diabetes and prediabetes [45]. This could explain why our observed IFG prevalence is lower than the prediabetes prevalence among Taiwanese adults.

Table 2 and Table 3 found that female participants sustained high neck and shoulder pain risk compared to male participants (*p* = 0.018; B = 0.16, *p* = 0.06). Previous research demonstrated that females more frequently reported neck, shoulder, waist, or back pain compared to males [46]. This could be due to the primary male sex hormone, testosterone, which provides protection for men against chronic MS pain conditions [47].

Regarding the relationship between daily coffee consumption and MS pain, previous studies have determined that individuals who consume high amounts of caffeine experience more severe pain than those who consume lower amounts [48]. Table 3 demonstrates that, in comparison to our study, individuals who consume at least one cup of coffee per day had a higher risk of neck and shoulder pain than others (B = 0.13, *p* = 0.03). This trend was consistently observed. However, it is important to note that this study does not establish that coffee intake induces MS pain, nor does it exclude the possibility that work-related MS pain may lead workers to increase coffee consumption, due to the limitations of our cross-sectional data collection.

The relationship between alcohol consumption and pain was curvilinear [49]. Excessive alcohol use could lead to chronic pain by increasing the risk of traumatic injuries and harming the MS system [50]. Moreover, alcohol exhibits potent, dose-dependent analgesic effects, which may contribute to self-medication behaviors in pain patients [51]. This may strengthen the bidirectional association between alcohol and MS pain. Our study corroborates the findings of other research. Table 2 shows a significant relationship between the frequency of alcohol use in the past month and the risk of neck and shoulder (*p* < 0.001) or ankle pain (*p* = 0.002). Additionally, Table 3 indicates that individuals who consumed alcohol in the past month had a higher risk of neck and shoulder pain compared to those who did not consume alcohol (B = 0.23, *p* < 0.001).

Notably, chronic pain is an important risk factor for sleep disorders. The two often occur simultaneously, exacerbating each other and forming a vicious cycle [52]. A study of middle-aged Americans showed that sleeping less than 6 h is linked to increased pain the next day [53]. Sleep loss initially triggers inflammation through monocytes, macrophages, and neutrophils, then shifts toward allergic and autoimmune responses (Th2/Th17). This increases pain signals (IL-6, IL-1β, TNFα) while reducing pain relief (IL-2), potentially heightening pain—such as in the neck and shoulders—or delaying recovery [54]. Additionally, pain intensity predicted decreased sleep quality the following night, and conversely, poor sleep quality predicted increased pain the following day [55]. Our study found a similar result: people who report sleeping less than 6 h per day sustained a higher risk of neck and shoulder pain than those who report sleeping more than 6 h per day (Table 3, B = 0.14, *p* = 0.020).

Previous studies found that MS pain is associated with daily work hours [56] and was a common risk factor for MS pain [16], too. Table 3 further provides evidence of the effect of work hours on the risk of neck and shoulder pain (B = 0.08, *p* = 0.017).

Table 2 found that MS pain caused by IFG is specifically localized to the neck and shoulders. The risk of neck and shoulder pain was higher in individuals with IFG compared to those without IFG (*p* = 0.030). IFG was identified as an independent risk factor for neck and shoulder pain (Table 3, B = 0.19, *p* = 0.005). The link between prediabetes and MS pain may arise from the following physiological pathways: hyperglycemia and the production of reactive species can directly increase pain signaling and activate sensory neurons, with these effects being possibly mediated by mitochondrial damage and increased inflammation [57]. A study in 2009 indicated that persistent chronic pain in multiple areas is an additional symptom of prediabetes and diabetes [58], and a cross-sectional survey in Saudi Arabia found that people with diabetes or prediabetes often experience chronic pain in the lower limbs, back, and neck, with the prevalence being 1.93 times higher than individuals without diabetes or prediabetes (OR = 1.93, *p* = 0.037) [59].

Previous research provides insights into the potential physiological relationship between hyperglycemia and MS pain. Obese individuals may be in a state of low-grade inflammation, which can induce long-term insulin resistance [60] and eventually lead to hyperglycemia [61]. Further, the accumulation of glycation end products in the connective tissues and joints is particularly likely to cause pain in the neck, knees, hips, shoulders, back, and arms [24]. This highlights the role of IFG in MS pain and led us to consider whether non-obese individuals with IFG have an increased risk of MS pain. Our research findings confirm this possibility. Table 3 shows that, in stratified analysis, IFG was an independent risk factor for neck and shoulder pain in individuals who were not overweight or obese (B = 0.29, *p* = 0.003). Notably, IFG was not associated with neck and shoulder pain risk in the presence of adjusted variables in individuals who were overweight or obese (B = 0.08, *p* = 0.444). Do the results in Table 3 refute the effect of IFG on MS pain? From a broader perspective, the obesity-related pain mechanisms are mechanical, behavioral, and physiological. For instance, carrying excess weight exerts mechanical pressure on the intervertebral discs, weight-bearing joints, and skeletal muscles. Sleep and physical activity are the two factors believed to influence the behavioral mechanisms underlying pain and obesity [29], which indicates that the relationship between obesity and MS pain is influenced by numerous non-physiological risk factors. Regrettably, these factors were beyond the scope of our investigation, preventing us from directly identifying their association. However, the mediation analysis enabled us to further investigate this relationship from an indirect influence perspective. Figure 1 confirms that IFG fully mediates ($Z_{m}=2.57,\;p<0.05$) the relationship between being overweight or obese and elevated neck and shoulder pain risk. This implies that individuals who are overweight or obese are more likely to suffer from IFG, which would sustain a higher risk of neck and shoulder pain, compared to those who are healthy weight or underweight. These findings provide strong evidence for the effect of IFG on musculoskeletal pain, particularly in the neck and shoulders.

## 5. Limitations

Our research did not examine whether the causes of MS pain were work-related or due to physical factors. This limitation could influence the findings regarding the impact of being overweight and obese on musculoskeletal pain. Moreover, MS pain can result from workload, work styles, or posture. Unfortunately, our study did not incorporate such data into the regression models. This limitation prevents us from adjusting for the effects of other confounders on MS pain in multiple linear regression models. Since the mediation model of an observational study can be biased [62], establishing ‘causal relationships’ carries a higher risk of misjudgment. Therefore, we refrain from concluding a ‘causal relationship’ and instead use ‘indirect effect’ in our conclusion to avoid misleading readers. Moreover, mediation analysis is a valid statistical tool for exploring such associations in cross-sectional data; the lack of temporal sequencing in our design precludes causal inference. For instance, it remains unclear whether IFG precedes pain onset, or if pain-related factors (e.g., reduced physical activity) contribute to glucose dysregulation. Thus, these results should be viewed as hypothesis-generating, supporting the need for longitudinal studies to test the directionality and causality of this pathway.

Additionally, although we adjusted for sex effects in the model, the participants were predominantly female healthcare workers (85.18%), which could affect the final results as we did not use sampling analysis for sex. Therefore, we will highlight ‘female-dominated healthcare workers’ in the conclusions.

Notably, we used the standards for overweight and obesity set by the Health Promotion Administration, Ministry of Health and Welfare, Taiwan. Therefore, the BMI thresholds for overweight and obesity in our study may be lower than those established by the WHO. Therefore, in our conclusions, we use the term ‘increased body weight’ rather than ‘overweight and obese’. We avoided using ‘prediabetes’ in our study’s results and conclusions, opting for ‘IFG’ instead, because we did not collect impaired glucose tolerance data. This term more accurately reflects the actual conditions.

One limitation is that variables labeled as ‘independent risk factors’ reflect statistical significance in our multivariable regression model, not confirmed causality. Future longitudinal or experimental studies may be needed to establish these factors’ causal roles.

Prediabetes, as defined in this study, is characterized by elevated blood glucose levels that exceed the normal range but fall below the diagnostic threshold for diabetes. This condition is strongly associated with an increased risk of progression to type 2 diabetes (American Diabetes Association, 2023) [63]. Type 1 diabetes, an autoimmune disorder characterized by acute onset and distinct etiological mechanisms, is excluded from the scope of this discussion. Consequently, the findings of this study should not be extrapolated to type 1 diabetes.

## 6. Conclusions

Among female-dominated healthcare workers, IFG, consuming at least one cup of coffee per day, drinking alcohol in the past month, sleeping less than 6 h daily, suffering from chronic diseases (excluding diabetes), and increased daily work hours were all independent risk factors for neck and shoulder pain. Notably, IFG was significantly correlated with neck and shoulder pain, with this association observed across individuals with and without increased body weight. These findings suggest that maintaining healthy blood glucose levels may be linked to lower musculoskeletal pain risk, though further longitudinal studies are needed to clarify the directionality and causality of this relationship.

## Figures and Tables

**Table 1:** The Nordic Musculoskeletal Questionnaire Musculoskeletal pain sites and factor analysis for 1039 participants.

Musculoskeletal Pain Sites	MS Pain Risk ^1^		Factor Loading ^2^	
	Mean	SD	Factor 1	Factor 2
**Neck** ^3^	517.85	1010.84	0.33	−0.01
**Left shoulder** ^3^	329.98	933.66	0.32	−0.08
**Right shoulder** ^3^	348.87	905.24	0.30	0.00
Upper back	270.40	906.58	0.15	−0.03
Waist or lower back	538.14	1180.10	0.08	−0.04
Left elbow	57.65	440.66	−0.06	−0.14
Right elbow	107.51	624.74	−0.01	−0.01
Left wrist	89.17	537.78	−0.02	0.07
Right wrist	196.87	754.77	−0.01	0.18
Left hip/thigh/buttock	84.84	664.35	−0.03	−0.18
Right hip/thigh/buttock	88.07	615.27	−0.03	0.02
Left knee	91.48	575.78	0.00	−0.05
Right knee	74.49	483.41	−0.06	−0.01
**Left ankle** ^3^	56.79	459.45	−0.02	0.57
**Right ankle** ^3^	43.19	354.38	−0.02	0.37
Eigenvalues			6.06	1.83
Explained variation accumulation of (%)			63.87	83.16

^1^ This is a product of the frequency score multiplied by the serious degree score. ^2^ The factor loading of eigenvalues more than 1. ^3^ Bold fonts represent pain sites with relatively large factor loadings.

**Table 2:** Description of demographic, survey variables, and MS pain for individuals.

Surveyed Variable	N (%/m ± SD)	MS Pain Risk			
		Neck and Shoulder	*p*	Both Ankles	*p*
IFG					
with	220 (21.17)	0.17 ± 1.36	0.030 ^a^	0.06 ± 1.01	0.265 ^a^
without	819 (78.83)	−0.04 ± 0.76		−0.02 ± 0.74	
Sex					
female	885 (85.18)	0.02 ± 0.97	0.018 ^a^	0.01 ± 0.85	0.430 ^a^
male	154 (14.82)	−0.12 ± 0.63		−0.03 ± 0.46	
Age					
age	1039 (37.50 ± 9.95)	0.03	0.349 ^c^	0.05	0.080 ^c^
Married					
Yes	453 (43.60)	0.04 ± 1.1	0.218 ^a^	0.01 ± 0.81	0.626 ^a^
Other	586 (56.40)	−0.03 ± 0.77		−0.01 ± 0.80	
Coffee drinking habits					
never	169 (16.27)	−0.14 ± 0.62	0.014 ^b^	−0.10 ± 0.22	0.011 ^b^
occasionally	456 (43.89)	−0.05 ± 0.86		−0.02 ± 0.73	
one cup per day	355 (34.17)	0.11 ± 1.12		0.03 ± 0.80	
two cups per day	45 (4.33)	−0.01 ± 0.64		0.37 ± 2.08	
at least two cups per day	14 (1.35)	0.39 ± 1.13		−0.18 ± 0.17	
Alcohol use in the past month					
never	653 (62.85)	−0.01 ± 0.72	<0.001 ^b^	−0.01 ± 0.83	0.002 ^b^
occasionally	383 (36.86)	0.15 ± 1.18		0.001 ± 0.72	
daily	3 (0.29)	1.10 ± 1.39		1.61 ± 3.12	
Daily sleeping time					
<5 h	47 (4.52)	0.25 ± 1.00	0.008 ^b^	−014 ± 0.45	0.312 ^b^
5–6 h	377 (36.28)	0.10 ± 1.14		0.05 ± 1.12	
6–7 h	444 (42.73)	−0.06 ± 0.78		−0.03 ± 0.51	
7–8 h	143 (13.76)	−0.16 ± 0.63		0.04 ± 0.71	
>8 h	28 (2.69)	−0.03 ± 0.80		−0.13 ± 0.18	
Exercise habits					
never	65 (6.26)	0.17 ± 1.75	0.024 ^b^	−0.12 ± 0.75	0.605 ^b^
less than once a month	249 (23.97)	0.12 ± 1.00		0.03 ± 0.96	
at least once a month	191 (18.38)	−0.02 ± 0.70		−0.002 ± 0.58	
at least once a week	464 (44.66)	−0.05 ± 0.82		0.01 ± 0.86	
at least once a day	70 (6.74)	−0.19 ± 0.67		−0.09 ± 0.30	
Body weight					
underweight	88 (8.47)	−0.16 ± 0.49	0.094 ^b^	−0.002 ± 0.45	0.534 ^b^
healthy weight	616 (59.29)	−0.02 ± 0.98		−0.03 ± 0.78	
overweight	192 (18.48)	0.12 ± 1.02		0.02 ± 0.78	
obesity	143 (13.76)	−0.05 ± 0.71		0.08 ± 1.06	
Chronic diseases (excluded diabetes)					
Yes	389 (37.44)	0.15 ± 1.19	<0.001 ^a^	0.05 ± 1.01	0.135 ^a^
No	650 (62.56)	−0.09 ± 0.71		−0.03 ± 0.66	
Profession fields					
Physician	121 (11.65)	0.07 ± 0.03	0.067 ^b^	0.03 ± 0.68	0.835 ^b^
Nurses	475 (45.72)	0.05 ± 0.83		−0.004 ± 0.82	
Technical staff	102 (9.82)	−0.20 ± 0.51		−0.06 ± 0.15	
Administration staff or others	341 (32.82)	−0.03 ± 0.98		0.02 ± 0.94	
Work hours					
Work time daily (h)	1039 (8.52 ± 0.88)	0.09	0.005 ^c^	0.03	0.357 ^c^
Shift work					
irregular shift	158 (15.21)	0.16 ± 1.29	0.153 ^b^	−0.10 ± 0.56	0.061 ^b^
regular shift	132 (12.70)	−0.03 ± 0.80		0.14 ± 1.19	
night shift	133 (12.80)	−0.04 ± 0.69		−0.08 ± 0.28	
day shift	616 (59.29)	−0.03 ± 0.88		0.01 ± 0.83	
Education degree					
PhD.	9 (0.87)	0.40 ± 1.21	0.119 ^b^	0.29 ± 1.21	0.725 ^b^
Master	137 (13.19)	0.15 ± 1.26		−0.02 ± 0.78	
Bachelor	856 (82.39)	−0.03 ± 0.86		−0.002 ± 0.80	
Others	37 (3.56)	−0.03 ± 0.68		0.04 ± 0.98	

N, individuals; m, mean value; SD, standard deviation; ^a^, *t*-test; ^b^, one-way ANOVA; ^c^, Pearson correlation analysis; *p*, *p* value.

**Table 3:** The multiple linear regression models for neck and shoulder pain risk and a stratified body weight analysis.

Surveyed Variable (Individuals)	Neck and Shoulder Pain Risk								
	All Participants			Without OW/OB			With OW/OB		
	B	SE	*p*	B	SE	*p*	B	SE	*p*
IFG									
with	0.19	0.07	0.005	0.29	0.10	0.003	0.08	0.10	0.444
without	1.00			1.00			1.00		
Sex									
female	0.16	0.08	0.06	0.20	0.12	0.100	0.13	0.12	0.259
male	1.00			1.00			1.00		
Drinking coffee habits									
at least one cups per day (414)	0.13	0.06	0.03	0.17	0.07	0.015	0.04	0.10	0.709
never or occasionally (625)	1.00			1.00			1.00		
Alcohol use in the past month									
ever (386)	0.23	0.06	<0.001	0.23	0.07	0.001	0.20	0.10	0.047
never (653)	1.00			1.00			1.00		
Sleeping time every day									
≤6 h (424)	0.14	0.06	0.020	0.11	0.07	0.131	0.19	0.10	0.057
>6 h (615)	1.00			1.00			1.00		
Exercise habits									
at least once a week (534)	−0.11	0.06	0.051	−0.11	0.07	0.132	−0.12	0.10	0.229
less than once a week (505)	1.00			1.00			1.00		
Chronic diseases (excluded diabetes)									
Yes	0.20	0.06	<0.001	0.19	0.07	0.008	0.21	0.10	0.036
No	1.00			1.00			1.00		
Work time									
Work time daily (h)	0.08	0.03	0.017	0.07	0.04	0.100	0.09	0.05	0.071

B, linear regression coefficient; SE, standard error; *p*, *p* value; OW/OB, overweight/obesity.

## Data Availability

The datasets used and/or analyzed during the current study are available from the corresponding author on reasonable request.
